# Challenges in Diagnosing Uterine Leiomyosarcoma in Pregnancy: A Case Report and Lessons Learned

**DOI:** 10.7759/cureus.94530

**Published:** 2025-10-14

**Authors:** Maryam Fatima, Chameli Subbaraj, Mustafa Guma

**Affiliations:** 1 Obstetrics and Gynecology, Basildon University Hospital, Basildon, GBR

**Keywords:** degenerating leiomyoma, malignant neoplasm, metastasis, myomectomy, uterine leiomyosarcoma

## Abstract

Uterine leiomyosarcoma (uLMS) is a rare, highly malignant neoplasm arising from the smooth muscle of the uterus and is exceedingly rare in pregnancy. We present a unique case highlighting the risk of misdiagnosis of uLMS as the more common condition of benign degenerating leiomyoma in pregnancy. Our case involves a 35-year-old, primiparous woman, where on a third-trimester scan (30 weeks), a high index of suspicion was raised for uLMS in a pre-existing benign leiomyoma. However, subsequent MRI assessments and multidisciplinary team (MDT) discussions inaccurately attributed it to the degeneration of a fibroid. uLMS was only diagnosed on histopathological examination when she underwent myomectomy for the presumed fibroid eight months postpartum. This caused a significant delay in the definitive surgical management and contributed to substantial iatrogenic upstaging of the disease (i.e., widespread metastasis of parametrial extension, pelvic lymphadenopathy, and pulmonary metastases), ultimately necessitating palliative second-line chemotherapy. Our case report aims to enhance clinicians’ understanding of the atypical clinical and radiological features of uLMS with improved detection and management.

## Introduction

Uterine leiomyosarcomas (uLMS) are smooth muscle tumors of monoclonal origin arising from the smooth muscle wall of the uterus [1]. It is hypothesized and demonstrated that uLMS originate from the abnormal stem cells of the myometrium; however, the specific cell of origin is yet to be identified [1]. It has a global incidence of approximately 0.64 per 100,000 women per year [2]. It typically arises de novo from the myometrium, with only 0.2% arising from pre-existing leiomyoma [3]. Most commonly, LMS presents after childbearing age, and the reported age of patients ranges from 45.0 to 56.9 years old. Occurrence during childbearing age is not common, and uLMS during pregnancy is even rarer, with only a few cases reported thus far in medical literature [4].

The clinicopathological and imaging characteristics of uLMS and benign uterine leiomyoma considerably overlap in pregnancy. However, the management of each diagnosis is vastly different [1]. Uterine leiomyomas are benign and are treated based on the degree and severity of symptomatology, while uLMS are tumors of high malignant potential, particularly notorious for early hematogenous spread, poor prognosis, and increased recurrence rates [3]. Earlier diagnosis and definitive surgical treatment are the mainstay of management for an optimized outcome. This case report adequately explains the atypical features of uLMS, which should alert clinicians and assist in addressing diagnostic challenges and therapeutic dilemmas associated with this rare malignancy.

## Case presentation

**Figure 1:**
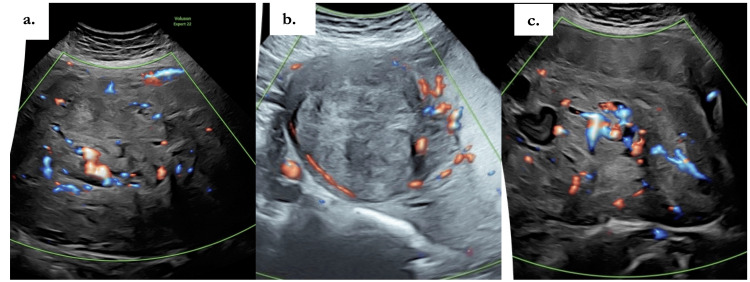
Color Doppler ultrasound in the third trimester. a: overview of a large, heterogeneous uterine mass displacing the uterus; b: peripheral vascularity along the lesion margin; c: abundant central intratumoral vascularity concerning for sarcoma.

This is a case of a 35-year-old White British primiparous woman who conceived spontaneously and was initially categorized as a low-risk pregnancy, with no significant past medical or surgical history. At her routine 12-week scan, a viable intrauterine pregnancy was confirmed, and two large intramural fibroids were incidentally noted in the anterior uterine wall, predominantly in the lower segment. The largest fibroid measured 17 cm. The 20-week anomaly scan was otherwise normal, and no concerns were raised in respect of the previously observed fibroid. She was followed under consultant-led care with increased surveillance and serial scans. At 28 weeks, ultrasound revealed a growth-restricted fetus (estimated fetal weight (EFW) < 5th centile) with normal amniotic fluid volume and Doppler studies. Fibroid measurements were not taken due to maternal discomfort from supine hypotension and breathlessness. A referral was made to the Fetal Medicine Unit (FMU) for suspected fetal growth restriction (FGR). At 30 weeks, an FMU scan confirmed FGR (EFW < 5th centile) with normal liquor volumes and Dopplers (Figure 1a). Additionally, it identified a large, highly vascular uterine mass measuring 148 mm × 208 mm × 176 mm in the left lateral wall. Unlike typical fibroids, which normally demonstrate peripheral vascularity, this mass showed abundant central vascularity, was largely solid with areas of cystic components, and was displacing the uterus and fetus superiorly (Figure 1a-1c). These findings correlated with her increasing symptoms of dyspnea when supine.

Given the features concerning malignancy, an urgent gynae review was recommended, as well as further imaging, an MRI. An urgent MRI was performed at 32 weeks. On T1-weighted images, the lesion demonstrated pockets of high signal intensity, consistent with areas of hemorrhagic degeneration. On T2-weighted sequences, the mass appeared heterogeneous with predominantly intermediate signal intensity. The mass was concluded to be a large subserosal fibroid (198 mm × 140 mm × 225 mm) in the anterior lower segment with pockets of hemorrhagic degeneration and central vascularity. No other abdominal or pelvic abnormalities were identified. Although the radiologist recommended a multidisciplinary team (MDT) discussion due to atypical features, the mass was still considered low risk for malignancy because it had not demonstrated significant interval growth. At 32+4 weeks, a repeat FMU scan showed static fetal growth over two weeks with signs of redistribution. The mass, now measuring 225 × 141 × 210 mm, was extensively vascular and occupied nearly the entire pelvis, extending to the umbilicus. Delivery was planned according to the scan's findings, by cesarean section after fetal optimization was achieved with antenatal corticosteroids and magnesium sulfate. A midline incision and classical uterine incision were proposed on the FMU report due to the large lower-segment mass displacing the fetus upwards, and for the highly vascular mass not to be disturbed.

At 33+2 weeks, she underwent a category 3 cesarean section via classical incision. Intraoperatively, a massive subserosal mass, considered a fibroid, covered by the uterovesical peritoneal fold, was noted in the lower segment, extending to the umbilicus. It appeared soft in consistency and was presumed to be degenerating. Blood loss was estimated at 1200 mL. Postoperative recovery was uneventful, and awaiting MDT discussion. As recommended antenatally, the case was reviewed at MDT one week postpartum. The consensus diagnosis was benign fibroid degeneration, and surveillance MRI was advised at three months. The follow-up MRI showed an interval reduction of the mass from 22 cm to 13 cm, though its overall features remained concerning. Discussion at MDT again supported the diagnosis of benign degeneration, as regression in size was considered atypical of sarcoma. After discussion with the patient, an open myomectomy was planned. She did not report any significant symptoms and was essentially amenorrheic during this time.

Approximately eight months postpartum, an open myomectomy was performed. A large subserosal fibroid extending into the left broad ligament was excised. An additional mass at the level of the cervix, infiltrating the posterior peritoneum, was noted and biopsied. The rest of the abdomen and pelvis appeared grossly normal. Histopathological analysis, however, confirmed leiomyosarcoma, demonstrating atypical spindle-to-epithelioid cells with marked nuclear pleomorphism, hyperchromasia, atypical mitoses, and extensive necrosis. Staging MRI and CT abdomen/pelvis revealed a residual 12 cm malignant pelvic mass compressing the left ureter, resulting in left hydronephrosis, along with extensive pelvic lymphadenopathy and probable posterior peritoneal infiltration. A PET scan confirmed multifocal pulmonary metastases.

The case was referred to a regional sarcoma MDT, and the consensus was to proceed with cytoreductive surgery followed by adjuvant chemotherapy. She underwent a total abdominal hysterectomy, bilateral salpingo-oophorectomy, sigmoid resection with stoma formation, and left ureteric resection with reimplantation and stenting. Histology confirmed high-grade leiomyosarcoma involving the myometrium with extension into the parametrial margin and subserosa of the colon (40 mm clear margin) and encompassing the left ureter (margin free of tumor). Postoperative imaging confirmed pulmonary, peritoneal, and liver metastases. Despite three cycles of first-line chemotherapy, the disease progressed, and she is currently receiving second-line systemic chemotherapy.

## Discussion

uLMS represent only 1-2% of uterine malignancies, yet they account for a disproportionate number of uterine cancer-related deaths due to their aggressive clinical course [1]. In contrast, uterine leiomyomas are the most common benign uterine tumors and are generally associated with an excellent prognosis. Distinguishing uLMS from leiomyomas is therefore of paramount importance, as the therapeutic approach and outcomes differ drastically.

On ultrasound, leiomyomas are usually characterized by a relatively hypovascular core with predominant peripheral vascularity. By comparison, uLMS often demonstrates abundant central vascularity, which can serve as a useful differentiator (Figure 1c) [5]. MRI provides superior soft-tissue characterization and is regarded as the most reliable modality for evaluating myometrial masses. Non-degenerated leiomyomas typically appear as circumscribed, low-signal intensity lesions on both T1- and T2-weighted sequences. However, large, degenerated fibroids, particularly those with red degeneration or hemorrhagic necrosis, can closely mimic the radiological features of uLMS [6]. Lakhman et al. have suggested that the presence of three or more MRI features, including irregular borders, T1 hyperintensity (hemorrhage), T2 dark areas, central unenhanced regions, disrupted endometrium, and restricted diffusion, improves the sensitivity and specificity of uLMS diagnosis to 95-100%, whereas degenerated leiomyomas typically exhibit only one or two of these features [7]. Despite this, overlap in imaging findings often results in diagnostic uncertainty, particularly during pregnancy.

The clinical presentation of uLMS is equally non-specific, commonly manifesting as a rapidly enlarging pelvic mass, abnormal uterine bleeding, or pelvic pain. During pregnancy, however, these symptoms are easily obscured by normal physiological changes, the enlarging uterus, and the well-documented tendency of benign leiomyomas to grow under hormonal influence. Moreover, the extreme rarity of uLMS in pregnancy fosters a natural diagnostic bias toward benign leiomyoma, often delaying appropriate intervention. The therapeutic dilemma arising from diagnostic uncertainty in such cases cannot be underestimated. Treatment of uLMS is inherently aggressive, typically involving total abdominal hysterectomy (TAH) with bilateral salpingo-oophorectomy (BSO) with or without adjuvant chemotherapy [8]. Erroneously diagnosing a young patient with uLMS would result in irreversible infertility with a profound impact on quality of life. However, not diagnosing uLMS has even more devastating consequences. In this case, multiple features pointed toward malignancy on imaging, including abundant central vascularity (a distinguishing marker from benign leiomyomas), together with high signal intensity on T1-weighted imaging, intermediate signal on T2-weighted imaging, and extensive hemorrhagic degeneration. Collectively, these features were worrisome. In contrast, the lack of significant interval growth, intraoperative gross findings consistent with degenerative leiomyoma, and marked postpartum regression of the mass all supported a benign etiology.

This case highlights the complexity of decision-making faced by the MDT, where suspicion of fibroid was maintained, likely due to the rarity of uLMS, the degenerative features observed radiologically and intraoperatively, and subsequent postpartum regression. In such a context, proceeding directly to aggressive surgery (TAH + BSO) was not a feasible option when benign degeneration remained a strong possibility. Nevertheless, given the concerning sonographic and MRI findings that prompted oncological discussion, consideration of myomectomy or biopsy at the time of cesarean section might have enabled earlier diagnosis and more tailored management. While it remains uncertain whether earlier diagnosis and treatment would have definitively altered prognosis, such an approach may have helped in slowing disease progression and, importantly, mitigating the medicolegal implications of delayed recognition. Unfortunately, the prognosis of uLMS remains poor, with a 5-year overall survival ranging from 25% to 76%, heavily dependent on stage at diagnosis [9]. Early hematogenous spread, most commonly to the lungs, liver, and peritoneum, and a high recurrence rate (up to 70% within two to three years) contribute to these poor outcomes [10]. Surgery, typically in the form of total hysterectomy with or without BSO, remains the mainstay of treatment. The role of adjuvant chemotherapy and radiotherapy is limited, with systemic therapy often used in a palliative setting.

## Conclusions

Overcoming the challenges involved in diagnosis and management requires a multimodal approach, requiring meticulous review of the imaging and correlating clinical presentations. This case report concludes that where there is a suspicion of uLMS, based on its clinical features like rapid growth or imaging findings like abundant vascularity, the possibility of uLMS must be strongly considered and should be excluded before attributing such findings to pregnancy-related changes. This will not only help to avoid the detrimental effects of widespread disease but will also contribute to mitigating the medicolegal aspects of missed diagnosis.
